# Microbubbles: from cancer detection to theranostics

**DOI:** 10.1186/1470-7330-15-S1-O17

**Published:** 2015-10-02

**Authors:** David Cosgrove

**Affiliations:** 1 Imaging Department, Hammersmith Hospital, Imperial College, London, UK; 2 Imaging Department, King's College Hospital, London, UK

## 

Microbubbles have proven to be useful as diagnostic agents for ultrasound[1,2]. However, they have the potential for a far wider range of uses, both in unmodified form (thus minimising regulatory barriers) and in new forms[3]. These future uses may be divided into diagnostic and therapeutic categories.

Routine diagnostic uses of microbubbles relevant to oncology are mainly in the liver, where they have been endorsed by NICE for the characterisation of focal liver lesions[4]. The key attribute here is the fact that malignancies do not have the functioning sinusoidal system that accounts for the late retention of microbubbles in the normal liver and in solid focal lesions - in this application, microbubbles are as effective as CT with contrast but less costly. Many similar oncological uses exploit microbubbles' exquisite ability to define both the macro- and microvasculature of tissue. Examples include characterising BI-RADS 3 and 4a breast masses, distinguishing pancreatic adenocarcinoma (hypoperfused) from focal pancreatitis (well perfused) and distinguishing complex-appearing real cysts from cystic carcinomas.

A potentially important extension is to develop targeted microbubbles that attach preferentially to cell surface molecules of interest[5]. Since conventional microbubbles of micron diameter cannot escape from the blood pool, the initial target is the blood vasculature. Ligands to VEGF1 can be attached to microbubbles; numerous preclinical studies have shown these to be effective ways to image malignant neovascularisation and the first human trial in prostate cancer has been completed[6]. A difficulty has been the relatively poor binding power of the targeted microbubbles, especially in the non-immunogenic form suitable for human use. The same strategies that are used for molecular imaging in nuclear medicine and MR can be deployed: improve the specific binding or wait for clearance of the unbound agent from the blood stream. Another approach makes use of the fast time resolution of ultrasound and tries to recognise which microbubbles are fixed and which are moving, thus enabling the bound population to be selectively imaged. Efforts have also been made to detect differences in the echoes from free versus bound microbubbles.

An important advance in allowing access to tissue beyond the endothelium is the development of nanodroplets, made by cooling and pressurising microbubbles - at around 200nm in diameter, they are able to cross the endothelium, especially where its is leaky[7]. Sonication with acceptable ultrasound intensity can then be used to reform the original microbubbles, with their ligands intact. This opens the way to imaging tissues beyond the endothelium.

The simplest approach to therapy employing microbubbles is to make use of their mechanical vibrations[8] and this has been used to accelerate thrombus breakdown to promote revascularisation of the middle cerebral artery[9].

An interesting approach uses low ultrasound frequencies and intensities along with microbubbles to ‘massage’ the endothelium or other surfaces (remote palpation), relying on the effect of acoustic radiation force impulses (ARFI) to move microbubbles along the ultrasound beam[10]. This allows temporary opening of tight endothelial junctions, for example to open the blood-brain barrier and improve the penetration of co-administered i.v. drugs.

Co-administration obviates some of the regulatory barriers to modified microbubbles and has been used to augment chemotherapy of pancreatic cancer with gemcitabine[11]. Further such trials are anticipated. Microbubbles might also be used to augment high intensity focussed ultrasound (HIFU) which would speed up the method, thus removing one of the main barriers to its wider use.

The most direct approach to treatment with microbubbles is to tag them with active drugs such as chemotherapeutic agents; breaking these microbubbles with high MI pulses releases the agent so that high concentrations can be achieved locally. This has been shown in small animals to minimise the cardiotoxicity of adriamycin in breast cancer[12]. This therapeutic avenue could be combined with the nanodroplets method and with remote palpation to access tumours.

Thus, the diagnostic and therapeutic possibilities for microbubbles are extensive; clinically useful approaches in oncology can be anticipated in the not too distant future.
